# Physical and chemical properties of Coarse Woody Debris submitted to the natural process of decomposition in a Secondary Atlantic Forest Fragment in Brazil

**DOI:** 10.1038/s41598-023-34526-9

**Published:** 2023-05-05

**Authors:** Paulo Henrique Villanova, Carlos Moreira Miquelino Eleto Torres, Laércio Antônio Gonçalves Jacovine, Angélica de Cássia Oliveira Carneiro, Fabiane Carvalho Ballotin, Bruno Leão Said Schettini, Samuel José Silva Soares da Rocha, Maria Paula Miranda Xavier Rufino, Mariany Filipini de Freitas, Renato Vinícius Oliveira Castro

**Affiliations:** 1 Departamento de Engenharia Florestal, Universidade Federal de Viçosa, Viçosa, Minas Gerais Brazil; 2 Departamento de Solos, Universidade Federal de Viçosa, Viçosa, Minas Gerais Brazil; 3 Departamento de Engenharia Florestal, Universidade Federal de Lavras, Lavras, Minas Gerais Brazil; 4 Departamento de Engenharia Florestal, Universidade Federal de São João del-Rei, Sete Lagoas, Minas Gerais Brazil

**Keywords:** Biogeochemistry, Biogeochemistry, Biogeochemistry, Carbon cycle

## Abstract

Coarse Woody Debris (CWDs) are constantly exposed to the natural decomposition process of wood, which can lead to a change in its physical–chemical properties. However, these changes have not yet been fully elucidated, requiring further studies to help to understand the effect of this process on CWDs degradation. Thus, the objectives of this study were: (i) verify if the decomposition affects the physical–chemical properties of the CWDs; (ii) verify if the structural chemical composition of the CWDs is altered as a function of decomposition, using immediate chemical and thermogravimetric analysis. Wood samples were collected from the CWDs to carry out these analyses, considering pieces with diameters ≥ 5 cm separated into 4 decay classes. The results indicated that the average apparent density decreased as a function of the increase of CWDs decomposition (0.62–0.37 g cm^−3^). The averages contents of Carbon and Nitrogen suffered less impact with the increase of CWDs decompositions, ranging from 49.66 to 48.80% and 0.52 to 0.58%, respectively. Immediate chemical and thermogravimetric analysis indicated a loss of holocelluloses and extractives and an increase in the concentration of lignin and ash throughout the decomposition process. The weight loss analyzed by thermogravimetric analysis was greater for less decomposed CWDs and with larger diameters. The use of these analyzes removes the subjectivity of CWDs decay classes, reducing the number of tests to determine CWDs physical–chemical properties and increasing the studies accuracy focused on the carbon cycle of these materials.

## Introduction

Coarse Woody Debris (CWDs) play important ecological role within the forest ecosystem^1–3^, serving as food for saprophagous organisms^4,5^, habitat for vertebrates and invertebrates^6^, and as a component of the carbon cycle^7–9^. The relevance of CWDs in the carbon cycle has expanded over the years due to the tree mortality increase^10^. Factors such as forest degradation^11,12^, land use change^13,14^ and the negative effects of climate change^15,16^ are primarily responsible for this growth in tree mortality in forests around the world, including the forests of the Brazilian Atlantic Forest^17,18^.

The carbon contained in CWDs can be stored for over 30 years in tropical environments^19,20^. However, the decomposition process, makes this forest component an emitting source of carbon to the atmosphere^21–23^. The decomposition of CWDs is a process that involves a complex series of transformations, which leads to the reduction of organic structures to their mineral form^24^. During this process, the physical and chemical properties of CWDs are altered due to microbial action (respiration and biological transformation), insects, physical degradation, leaching and fire^1,25–27^.

Changes in the physical and chemical properties of CWDs begin with the degradation of wood cell walls, which are mostly made up of holocelluloses (cellulose and hemicellulose) and lignin^28,29^. The concentrations of these structural compounds in dead wood are modified as decomposition progresses^30^. The concentration of holocelluloses is preferentially reduced in the early stages of CWDs decomposition. In more advanced stages, the holocelluloses are selectively decomposed and lignin concentration tends to increase in the remaining materials^31–33^.

These modifications in holocelluloses and lignin concentrations due to the decomposition process affects the apparent density^34,35^ and the contents of chemical compounds such as Carbon (C) and Nitrogen (N) of the CWDs^36–39^. Furthermore, the concentrations of these structural compounds influence the CWDs resistance to decomposition^24,40^ and determine the degree of deterioration of wood already subjected to this process, removing the subjectivity in the classification of the decay class of these materials^1,25,38^. In this sense, chemical analyzes such as immediate analysis and thermogravimetric analysis, which indicates the structural chemical composition of CWDs from determination of volatile materials content, fixed carbon, residual weight (ash) and weight loss in different temperature ranges, become essential for understanding the effect of CWDs decomposition.

In our study, we present a physical and chemical characterization of CWDs submitted to the natural process of decomposition, which contributes to the understanding of the wood degradation dynamics in tropical forests. In addition, our study can be used to support the carbon stock quantification by CWDs in international reports such as the Forest Resources Assessments (FRA)^41^, which address the protection and sustainability of forest management in worldwide^42^.

Thus, the objectives of this study were: (i) to verify if the decomposition affects the physical properties (apparent density) and chemical properties (elemental chemical analysis) of the CWDs in a secondary forest of the Atlantic Forest; (ii) verify if the chemical structural composition of CWDs is altered as a function of the natural process of decomposition using immediate chemical analysis and thermogravimetric analysis (TG/DTG).

## Material and methods

### Characterization of the study area

**Figure 1:**
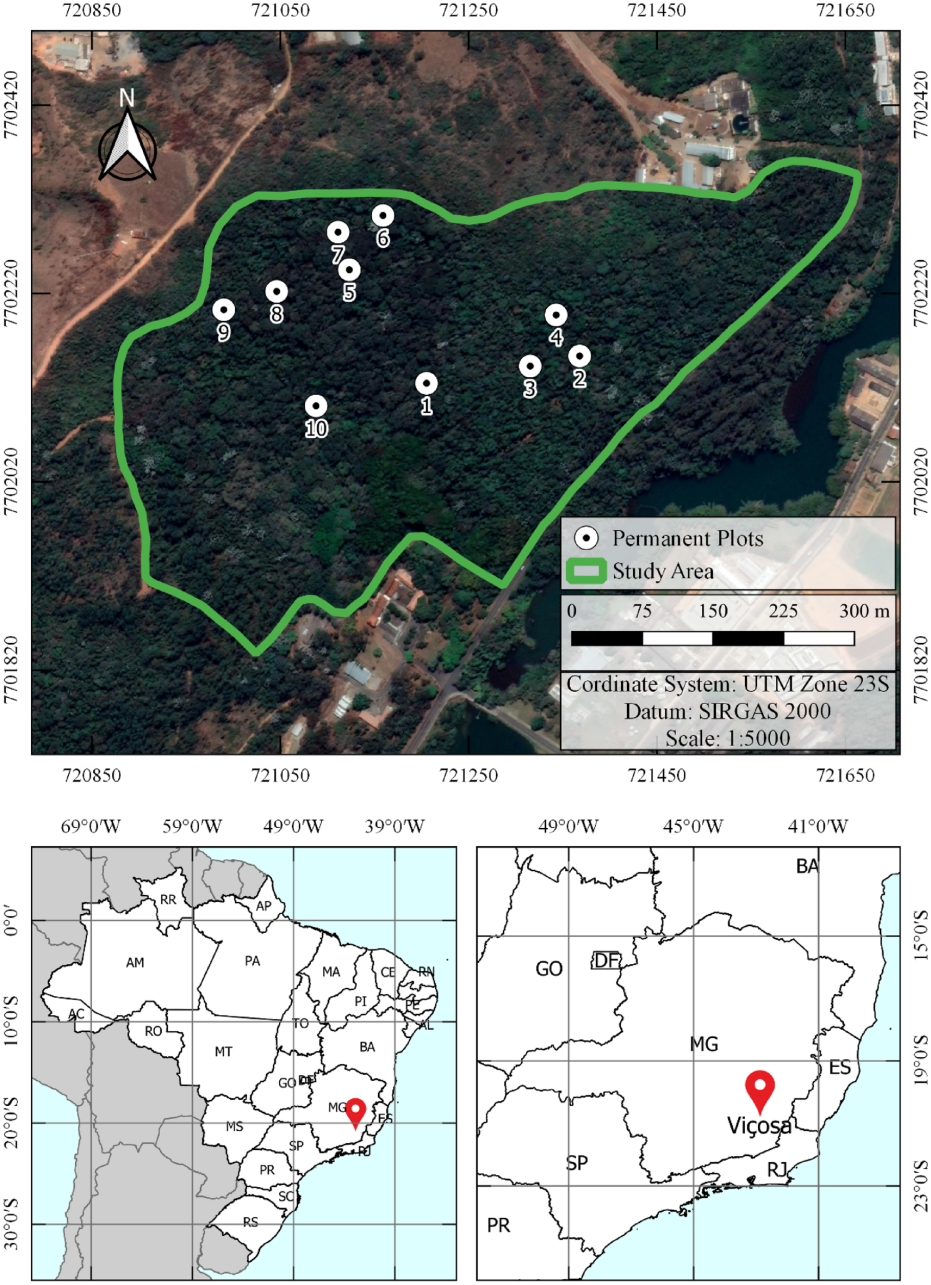
Location of the studied at Atlantic Forest fragment (the map was generated using QGIS 3.10.6-http://qgis.osgeo.org^45^.

The study was carried out in a fragment of secondary Atlantic Forest with an area of 17 ha, known as “Mata da Silvicultura”, which is located in the municipality of Viçosa-MG, Brazil (Fig. 1). The local climate is Cwa type (Köppen classification) with temperature, humidity and average precipitation of 19.9 °C, 79.9% and 1269.4 mm, respectively^43^. The fragment's altitude varies from 670 to 730 m^16^ and the region has pedogeomorphologic gradients with aluminum-rich dystrophic latosols at the tops of hills, colluvial ramps with shallow latosols and cambic horizon, while the bottoms of the groves present a predominance of epieutrophic cambisols rich in nutrients^44^.

The vegetation in the region is classified as seasonal semideciduous forest^46^. The forest fragment studied is in a medium stage of regeneration^16^, with quadratic mean diameter and total heigh of trees ranging between 10 and 20 cm and 5 and 12 m, respectively^47^. Phytosociological parameters, floristic composition, dendrometric variables and forest dynamics were periodically quantified over the years (Table 1).

**Table 1:** Phytosociological parameters, floristic composition, dendrometric variables and forest dynamics of “Mata da Silvicultura”.

Variable	Mensuration year							
	1994	1997	2000	2004	2008	2010	2013	2016
Botanical Families	50	49	49	48	47	47	45	46
Botanical Genera	104	103	107	107	111	111	107	113
Identified Species	144	143	146	144	158	156	149	168
Pioneers	18	16	16	14	15	15	15	17
Initial Secondary	75	76	76	75	77	75	74	82
Late Secondary	39	40	41	41	44	45	41	47
Non-Identified Species	12	11	13	14	22	21	19	22
Shannon-Weaver Index (H’)	4.08	4.04	4.02	3.99	4.01	3.99	3.95	4.11
Density (stems ha^−1^)	1,543	1,564	1,524	1,504	1,531	1,479	1,405	1,432
Quadratic mean diameter (cm)	14.40	14.80	15.07	15.17	15.48	15.69	16.31	16.16
Basal Area (m^2^ ha^−1^)	25.12	26.90	27.19	27.20	28.80	28.60	29.35	29.36
Minimum DBH (cm)	5.09	5.06	5.06	5.06	5.03	5.03	5.03	5.03
Arithmetic mean diameter (cm)	11.83	12.03	12.19	12.26	12.40	12.50	12.93	12.60
Maximum DBH (cm)	80.21	82.12	84.03	85.63	91.04	91.35	94.22	94.22
Minimum Height (m)	2.50	2.50	2.50	2.40	2.40	2.07	2.07	2.40
Average Height (m)	10.60	11.20	11.52	12.55	12.52	12.33	12.89	12.83
Maximum Height (m)	32.07	32.35	33.63	39.20	39.20	38.00	38.00	38.00
Recruited Stems (stems ha^−1^)	-	116	80	57	126	41	18	204
Mortality Stems (stems ha^−1^)	95	120	79	108	93	92	177	–

### Data collection

Coarse Woody Debris (CWDs) such as branches, stumps and fallen trees on the forest floor and with a diameter ≥ 5 cm were inventoried in ten permanent plots of 20 m × 50 m, from July to October 2018. The arithmetic means of the diameters measured at the ends of the residues was calculated to separate the material into diameters classes with an amplitude of 5 cm. The CWDs found into these plots were divided into 4 classes according to their stage of decomposition^48–50^: (i) Materials that have just fallen to the ground with leaves and bark intact; (ii) Materials similar to those of class “i”, but with the bark showing rotting or peeling; (iii) Materials with a high stage of decomposition and showing some resistance to being broken; (iv) Materials that are rotten, friable and without resistance to being broken.

#### Sampling and sample preparation

Three samples of each diameter and decay class were collected, when possible, in the ten inventoried plots. Two subsamples were taken from this material to determine the physical analysis (apparent density) and the rest of the material was dried in an oven with forced air circulation at ± 60 °C, until its dry weight stabilized, for the determination of chemical (elementary and immediate) and thermogravimetric (TG/DTG) analyzes.

The oven-dried material was picked and ground in a Willey-type knife mill (Thomas Scientific®). The crushed material was sieved using 40 mesh and 60 mesh granulometric sieves for elemental, immediate and thermogravimetric chemical analysis. The materials retained in the 60 mesh sieves were homogenized by randomly choosing three plots per diameter and decay class, totaling 28 composite samples for each type of analysis.

### Physical properties

#### Apparent density

The CWDs apparent density was determined through mercury immersion method due to the material fragility, according to NBR 11941^51,52^. The expression used to determine the apparent density was: $\rho = {\text{m}}/{\text{v}}$, where: ρ is the apparent density, in g cm^−3^; ${\text{m}}$ is the mass of the sample, in g; and ${\text{v}}$ is the apparent volume, in cm^3^. In this specific case, the apparent volume (v) was obtained by the expression: $\text{v } = {\text{m}}_{1}/{ \rho }_{\text{Hg}}$, where: m_1_ is the immersed mass, in g; and ρ_Hg_ is the density of mercury, in g cm^−3^. The mean values of apparent density were calculated for each diameter and decay class, considering the density arithmetic mean of each subsample.

### Chemical properties

#### Elementary chemical analysis

The Carbon (C) and Nitrogen (N) contents of CWDs were determined using a dry combustion elemental analyzer (LECO TruSpec® Micro Elemental Series CHN/CHNS/O; St. Joseph, MI). In this method, the gases emitted by samples burning at 1050ºC were quantified by an infrared detector, which allows the determination of the content, in %, of these elements^39^. The C/N ratio of CWDs was calculated as an indicator of the natural decomposition of these materials on the forest floor.

#### Immediate chemical analysis

Volatile materials (Vol), ash content (Ash) and fixed carbon (FC) were quantified using a Linn Elektro Therm® muffle furnace, according to the ASTM standards D1762-84^53^. The calculations to determine these parameters, in %, were based on the following expressions:

$$\text {Vol}=100 [(\text{P}_1 - \text{P}_2)/\text{P}],\text {Ash}=100[(\text{P}_2-\text{P}_0)/\text{P}] \text {e FC}=100-\text{Vol}-\text{Ash},$$

where P is the original mass of the sample, in g; P_0_ is the original mass of the crucible, in g; P_1_ is the initial mass of the crucible + the mass of the sample, in g; P_2_ is the final mass of the crucible + the mass of the sample, in g^54,55^.

#### Thermogravimetric analysis (TG/DTG)

The thermogravimetric analysis of CWDs was performed using a DTG-60H Shimadzu equipment, under a nitrogen atmosphere, with a constant flow rate of 50 ml min^−1^. Thermograms were obtained from a temperature of 100 °C to a temperature of 450 °C, with a heating rate of 10 °C min^−1^. The thermogravimetric curves (TG) of CWDs were analyzed by diameter and decay classes to evaluate the weight loss as a function of temperature, while the first derivative of the thermogravimetric curve (DTG) was obtained to identify the temperatures at which the highest weight losses occurred. Weight losses were calculated from TG curve for the following temperature ranges: 100–200 °C; 200–300 °C; 300–450 °C. The residual weight was obtained at a temperature of 450 °C, considering the mass of the absolutely dry sample at a temperature of 100 °C as the initial value.

### Statistical analysis

Analysis of Variance (ANOVA) and Tukey's post-hoc test was applied to test whether the means of the physical (apparent density) and chemical (elementary and immediate chemical analysis) parameters of the CWDs differed statistically between the decay classes. The Shapiro–Wilk test was performed to test the assumption of normality in the following thermogravimetric analysis datasets: (i) weight losses by decay classes of CWDs, in 3 temperature ranges; (ii) residual weight by decay classes of CWDs; (iii) residual weight by diameter classes of CWDs. Only the dataset with losses weight at temperatures of 100–200 °C and 200–300 °C violated the assumption of data normality (*P* < 0.05). In these cases, the nonparametric Kruskal–Wallis test was applied to test whether there were statistical differences between the medians of the evaluated groups. In the other data sets in which normality was reached (*P* > 0.05), Analysis of Variance (ANOVA), followed by Tukey's post-hoc test, were applied to test whether there were statistical differences between the means of the evaluated groups.

Spearman's correlation was calculated to quantify the association degree of two nonparametric variables in intensity and direction^16,56,57^. The correlation matrix was generated by the software R Core Team^58^ using the thermogravimetric, physical and chemical properties of CWDs.

### Complies with international, national and/or institutional guidelines

The collection of plant material, experimental research and field studies complied with relevant institutional, national, and international guidelines and legislation.

## Results

### Physical properties

#### Apparent density

The apparent density (mean ± standard deviation) decreased as a function of the decay class of CWDs, ranging from 0.62 g cm^−3^ (± 0.13 g cm^−3^) for the least decomposed residue (class 1) to 0.37 g cm^−3^ (± 0.17 g cm^−3^) for the most decomposed (class 4). The apparent average densities did not follow a pattern of increase or decrease in value as a function on the CWD’s diameter classes variation (Table 2).

**Table 2:** Apparent density (g cm^-3^) (mean ± standard deviation) by diameter and decay classes of CWDs.

Diameter Classes (cm)	Decay classes			
	1	2	3	4
7.5	0.60 ± 0.15	0.45 ± 0.16	0.38 ± 0.17	0.35 ± 0.17
12.5	0.65 ± 0.08	0.55 ± 0.18	0.34 ± 0.11	0.38 ± 0.15
17.5	–	0.44 ± 0.17	0.38 ± 0.18	0.35 ± 0.19
22.5	–	0.62 ± 0.09	0.41 ± 0.18	0.41 ± 0.23
27.5	–	1.05 ± 0.01	0.36 ± 0.12	0.37 ± 0.07
32.5	–	0.54 ± 0.01	0.55 ± 0.18	0.33 ± 0.06
37.5	–	–	0.49 ± 0.25	0.37 ± 0.04
42.5	–	–	0.42 ± 0.04	0.25 ± 0.16
47.5	–	–	0.68 ± 0.00	–
52.5	–	–	0.52 ± 0.01	–
57.5	–	–	0.72 ± 0.01	–
62.5	–	–	–	0.51 ± 0.03
Mean	0.62^a^ ± 0.13	0.50^a^ ± 0.18	0.39^b^ ± 0.17	0.37^b^ ± 0.17

Decay classes: (i) Materials that have just fallen to the ground with leaves and bark intact; (ii) Materials similar to those of class “i”, but with the bark showing rotting or peeling; (iii) Materials with a high stage of decomposition and showing some resistance to being broken; (iv) Materials that are rotten, friable and without resistance to being broken. Means followed by the same letter do not differ statistically by Tukey's test, at the 5% significance level.

### Chemical properties

#### Elementary chemical analysis

Carbon (C) contents (mean ± standard deviation) showed a low variation between the CWD decay classes: 49.66% (± 0.90) to 48.80% (± 1.39%). This same behavior was observed for Nitrogen (N) contents (mean ± standard deviation), which ranged from 0.44% (± 0.08%) to 0.58% (± 0.20%). The C/N ratio ranged from 88.14 (± 19.86) to 116.61 (± 21.72), with the lowest values for the highest decomposition classes. The CWD’s diameter classes did not significantly affect these parameters and they did not show a well-defined behavior pattern (Table 3).

**Table 3:** Carbon Contents-C (%) (mean ± standard deviation), Nitrogen-N (%) (mean ± standard deviation) and C/N Ratio (mean ± standard deviation) by diameter and decay classes of CWDs.

Diameter Classes (cm)	Decay classes											
	1			2			3			4		
	C	N	C/N	C	N	C/N	C	N	C/N	C	N	C/N
7.5	49.25 ± 0.07	0.60 ± 0.01	82.02 ± 1.18	49.95 ± 0.07	0.46 ± 0.00	107.54 ± 1.30	49.75 ± 0.21	0.95 ± 0.07	52.52 ± 3.57	48.70 ± 0.14	0.78 ± 0.01	62.16 ± 1.02
12.5	49.30 ± 0.00	0.43 ± 0.01	114.14 ± 2.24	49.40 ± 0.28	0.33 ± 0.02	150.03 ± 10.50	48.95 ± 0.21	0.77 ± 0.02	63.35 ± 1.81	50.20 ± 0.14	0.81 ± 0.00	61.98 ± 0.39
17.5	–	–	–	50.00 ± 0.42	0.56 ± 0.01	89.45 ± 0.60	49.20 ± 0.00	0.59 ± 0.01	83.54 ± 1.40	48.40 ± 0.14	0.71 ± 0.01	68.52 ± 1.10
22.5	–	–	–	48.30 ± 0.28	0.45 ± 0.03	108.14 ± 6.37	48.05 ± 0.07	0.43 ± 0.02	110.82 ± 4.86	48.25 ± 0.07	0.59 ± 0.01	82.08 ± 1.66
27.5	–	–	–	51.05 ± 0.07	0.37 ± 0.01	137.07 ± 2.53	49.25 ± 0.21	0.82 ± 0.03	60.27 ± 1.77	48.20 ± 0.14	0.50 ± 0.01	96.60 ± 1.65
32.5	–	–	–	49.25 ± 0.21	0.46 ± 0.01	107.44 ± 2.62	49.40 ± 0.00	0.51 ± 0.03	97.68 ± 5.32	48.15 ± 0.21	0.49 ± 0.02	99.08 ± 4.90
37.5	–	–	–	–	–	–	47.35 ± 0.07	0.64 ± 0.04	73.64 ± 4.09	50.40 ± 0.00	0.52 ± 0.02	96.32 ± 2.99
42.5	–	–	–	–	–	–	50.80 ± 0.00	0.56 ± 0.00	90.47 ± 0.57	46.25 ± 0.07	0.40 ± 0.02	116.02 ± 4.76
47.5	–	–	–	–	–	–	50.15 ± 0.21	0.32 ± 0.01	158.31 ± 6.32	–	–	–
52.5	–	–	–	–	–	–	48.60 ± 0.00	0.51 ± 0.01	94.30 ± 2.20	–	–	–
57.5	–	–	–	–	–	–	50.40 ± 0.42	0.29 ± 0.02	170.82 ± 8.78	–	–	–
62.5	–	–	–	–	–	–	–	–	–	50.65 ± 1.06	0.46 ± 0.00	110.48 ± 3.16
Mean	49.27^a^ ± 0.05	0.52^a^ ± 0.10	98.08^ab^ ± 18.60	49.66^a^ ± 0.90	0.44^a^ ± 0.08	116.61^a^ ± 21.72	49.26^a^ ± 1.00	0.58^a^ ± 0.20	95.98^ab^ ± 37.45	48.80^a^ ± 1.39	0.58^a^ ± 0.14	88.14^b^ ± 19.86

Decay classes: (i) Materials that have just fallen to the ground with leaves and bark intact; (ii) Materials similar to those of class “i”, but with the bark showing rotting or peeling; (iii) Materials with a high stage of decomposition and showing some resistance to being broken; (iv) Materials that are rotten, friable and without resistance to being broken. Means followed by the same letter do not differ statistically by Tukey's test, at the 5% significance level.

#### Immediate chemical analysis

The volatile materials (mean ± standard deviation) contained in the CWDs ranged from 67.71% (± 5.70%) to 81.59% (± 2.63%) while the ash content (mean ± standard deviation) showed values ranging from 1.47% (± 0.36%) to 10.07% (± 5.89). The CWDs Fixed Carbon contents ranged from 16.63% to 22.22%. The parameters of the immediate chemical analysis did not show a pattern of behavior as a function of the increase in the CWD’s diameter classes (Table 4).

**Table 4:** Volatiles-Vol (%) (mean ± standard deviation), Ash content-Ash (%) (mean ± standard deviation) and Fixed Carbon-C (%) (mean) by diameter and decay classes of CWDs.

Diameter Classes (cm)	Decay classes											
	1			2			3			4		
	Vol	Ash	FC	Vol	Ash	FC	Vol	Ash	FC	Vol	Ash	FC
7.5	80.97 ± 1.00	1.65 ± 0.50	17.38	78.46 ± 0.71	7.39 ± 7.05	14.14	72.13 ± 0.52	6.55 ± 0.80	21.31	70.62 ± 2.52	13.09 ± 1.31	16.29
12.5	80.00 ± 1.16	1.30 ± 0.09	18.69	83.26 ± 1.09	0.93 ± 0.00	15.81	74.87 ± 0.46	6.91 ± 0.35	18.22	69.86 ± 1.35	8.41 ± 1.16	21.73
17.5	–	–	–	80.77 ± 2.21	1.48 ± 0.02	17.75	77.55 ± 1.00	5.22 ± 0.41	17.22	68.65 ± 0.93	2.48 ± 0.27	28.86
22.5	–	–	–	82.88 ± 1.54	0.38 ± 0.31	16.74	80.39 ± 1.56	1.25 ± 0.00	18.35	72.55 ± 1.11	4.53 ± 0.22	22.92
27.5	–	–	–	85.05 ± 0.79	0.16 ± 0.08	14.78	75.50 ± 1.50	2.34 ± 0.01	22.16	71.85 ± 1.79	7.14 ± 2.10	21.02
32.5	–	–	–	79.13 ± 0.10	0.34 ± 0.01	20.53	77.71 ± 1.12	1.31 ± 0.09	20.98	69.68 ± 0.90	12.01 ± 2.02	18.30
37.5	–	–	–	–	–	–	78.46 ± 0.79	1.22 ± 0.06	20.32	54.81 ± 0.72	22.25 ± 0.97	22.94
42.5	–	–	–	–	–	–	77.67 ± 2.42	1.96 ± 0.10	20.37	61.33 ± 0.91	14.04 ± 0.61	24.63
47.5	–	–	–	–	–	–	85.83 ± 0.73	0.28 ± 0.05	13.89	–	–	–
52.5	–	–	–	–	–	–	86.16 ± 0.37	1.18 ± 0.38	12.66	–	–	–
57.5	–	–	–	–	–	–	86.57 ± 0.38	0.25 ± 0.20	13.21	–	–	–
62.5	–	–	–	–	–	–	–	–	–	70.02 ± 0.29	6.68 ± 0.39	23.30
Mean	80.49^a^ ± 1.10	1.47^a^ ± 0.36	18.04^ab^	81.59^a^ ± 2.63	1.78^a^ ± 3.41	16.63^a^	79.35^a^ ± 4.82	2.65^a^ ± 2.44	18.06^a^	67.71^b^ ± 5.70	10.07^b^ ± 5.89	22.22^b^

Decay classes: (i) Materials that have just fallen to the ground with leaves and bark intact; (ii) Materials similar to those of class “i”, but with the bark showing rotting or peeling; (iii) Materials with a high stage of decomposition and showing some resistance to being broken; (iv) Materials that are rotten, friable and without resistance to being broken. Means followed by the same letter do not differ statistically by Tukey's test, at the 5% significance level.

#### Thermogravimetric analysis (TG/DTG)

The wood degradation components occurred in a narrow temperature range, partially overlapping, where: (i) water loss (0–100 °C); (ii) degradation of hemicellulose (225–275 °C); (iii) cellulose degradation (275–375 °C); and (iv) lignin degradation (> 370 °C).

**Figure 2:**
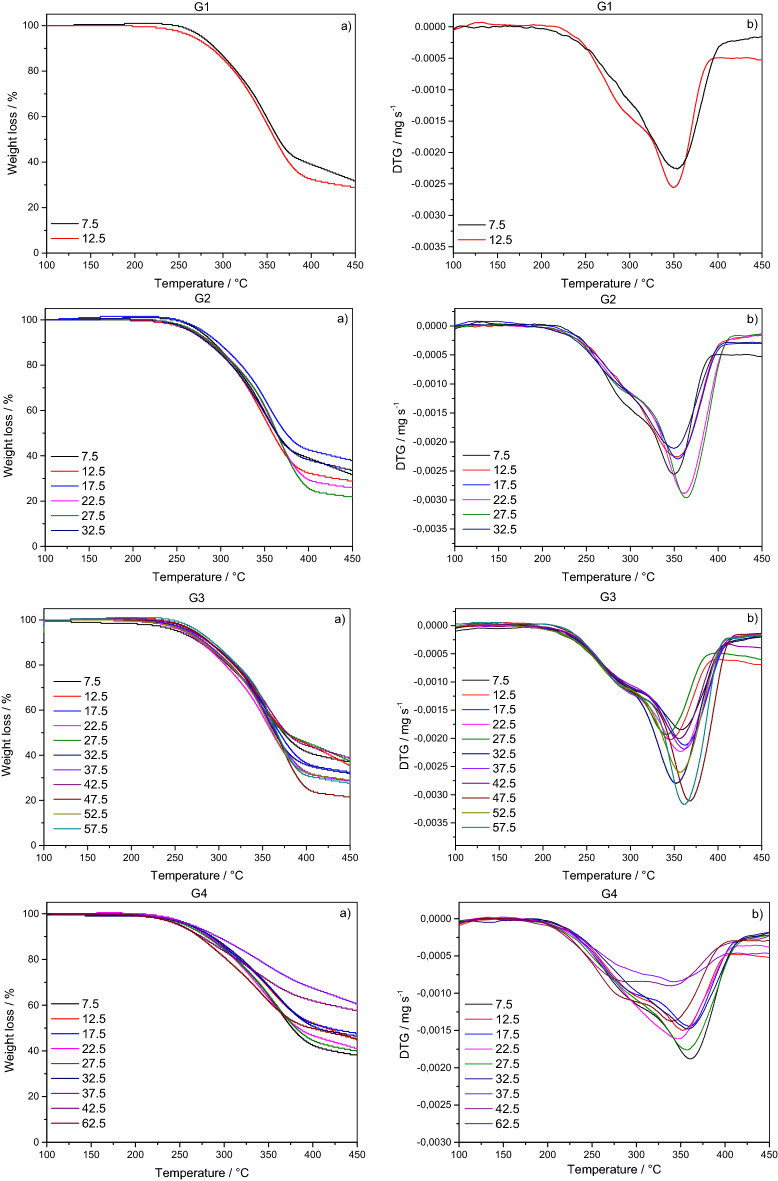
TG/DTG curves by diameter and decay classes of CWDs. (i) TG (**a**) and DTG (**b**) curve for decay class 1 (G1); (ii) TG (**a**) and DTG (**b**) curve for decay class 2 (G2); (iii) TG (**a**) and DTG (**b**) curve for decay class 3 (G3); (iv) TG (**a**) and DTG (**b**) curve for decay class 4 (G4). Decay classes: G1-Materials that have just fallen to the ground with leaves and bark intact; G2-Materials similar to those of class “i”, but with the bark showing rotting or peeling; G3-Materials with a high stage of decomposition and showing some resistance to being broken; G4-Materials that are rotten, friable and without resistance to being broken.

Thermogravimetric curves (TG/DTG) were obtained for each CWD diameter and decay class (Fig. 2). The curves indicated that thermal degradation profiles of CWDs suffered variations in the residual masses and in the maximum peaks of woods constituent’s degradations. Thermogravimetric curves (TG/DTG) indicated that thermal degradation profiles suffered variations in the residual masses and in the maximum peaks of woods constituent’s degradations according to the diametric and decomposition class of the CWDs. Weight losses for the first decay class (G1) were similar. However, a longer length of the DTG curve (close to 287 °C) was observed for the larger diameter sample. The second DTG peak (close to 351 °C) showed greater weight loss for samples with larger diameters. Weight losses for the second decay class (G2) were different, with smaller diameter samples showing greater length in the DTG curve (close to 295 °C). DTG peaks ranged from 348–361 °C, with larger diameters having higher peaks. The third decay class (G3) presented a wide range of weight losses, as well as in G2. The first DTG peak (close to 297 °C) did not show significant differences for the diameter classes of the CWDs. However, the maximum decomposition temperature of the second peak (close to 358–366 °C) increased as the CWD diameter increased. The last decay class (G4) showed a lower weight loss compared to the other decay classes. The DTG peaks (close to 277 °C) had a longer length for samples with larger diameters.

Weight losses and residual mass were quantified for each diameter and decay class of CWDs, in different temperature ranges (Table 5). The weight loss ranged from 0.00 to 1.55% in the first temperature range (100–200 °C), from 10.84 to 18.65% in the second temperature range (200–300 °C) and from 25.63 to 65.13% in the last temperature range (300–450 °C). The residual mass at 450 °C ranged from 21.54 to 60.59%, being higher in the more decomposed CWDs.

**Table 5:** Weight loss (%) as a function of temperature range and residual mass at 450 °C by diameter and decay classes of CWDs.

Decay classes	Diameter classes (cm)	Weight loss (%)			Residual weight (%)
		100–200 °C	200–300 °C	300–450 °C	
1	7.50	0.00	13.61	52.88	33.51
1	12.50	0.54	14.59	59.46	25.41
2	7.50	0.00	14.36	54.95	31.68
2	12.50	0.56	14.44	56.11	28.89
2	17.50	0.00	12.63	51.01	37.88
2	22.50	0.00	13.57	60.30	26.13
2	27.50	0.00	14.14	63.88	21.98
2	32.50	0.00	14.89	51.60	33.51
3	7.50	1.55	15.03	46.11	37.31
3	12.50	0.00	14.72	50.76	35.53
3	17.50	0.53	14.89	52.13	32.45
3	22.50	0.53	16.58	54.01	28.88
3	27.50	0.00	15.18	47.64	37.17
3	32.50	0.00	13.15	54.93	31.92
3	37.50	0.00	14.94	56.32	28.74
3	42.50	0.00	13.92	47.94	38.66
3	47.50	0.00	13.33	65.13	21.54
3	52.50	0.51	15.31	55.10	29.08
3	57.50	0.00	13.30	60.10	27.59
4	7.50	0.50	14.93	46.27	38.31
4	12.50	1.00	13.43	40.80	44.78
4	17.50	0.50	14.00	38.00	47.50
4	22.50	0.00	15.23	43.65	41.12
4	27.50	0.49	15.20	44.61	39.71
4	32.50	1.01	12.63	39.90	46.46
4	37.50	0.49	10.84	28.08	60.59
4	42.50	1.01	15.58	25.63	57.79
4	62.50	0.52	18.65	35.75	45.08

Decay classes: (i) Materials that have just fallen to the ground with leaves and bark intact; (ii) Materials similar to those of class “i”, but with the bark showing rotting or peeling; (iii) Materials with a high stage of decomposition and showing some resistance to being broken; (iv) Materials that are rotten, friable and without resistance to being broken.

#### Statistical analysis

**Figure 3:**
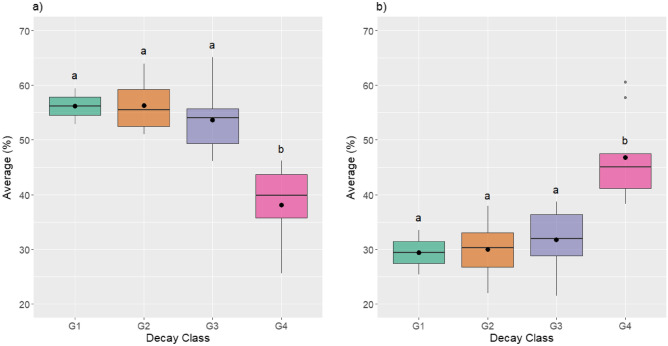
(**a**) Boxplot and weight loss averages, by decay class, in the temperature range of 300 – 450 °C; (**b**) Boxplot and residual mass averages at 450 °C, by decay class. Boxplots followed by the same letter do not differ statistically by Tukey's test, at the 5% significance level.

The Kruskal–Wallis test indicated that differences between weight loss medians in the first (100–200 °C) and second (200–300 °C) temperature ranges, by decay class, were not significant (*P* > 0.05). On the other hand, ANOVA indicated a significant difference in the range of 300–450 °C and for residual mass at 450 °C (*P* < 0.05), being the class 4 decomposition mean, different from the other classes by Tukey test (Fig. 3). In this way, the thermogravimetric analysis was able to differentiate CWD samples into two groups, the first involving decay classes 1, 2 and 3 and the second group involving only decay class 4. The differences between the residual mass averages, by CWD’s diameter classes, were not significant (*P* > 0.05).

In this way, the Spearman correlation was calculated for the weight losses in the temperature range of 300–450 °C and for the residual weight at 450 °C, separating the statistically different groups by the Tukey test into: Group 1 (G1)-decay classes 1, 2 and 3; Group 2 (G2)-decay class 4. The physical and chemical properties used to calculate the correlation were: volatile materials (%), ash content (%), fixed carbon (%), C/N ratio and apparent density (g cm^−3^).

**Figure 4:**
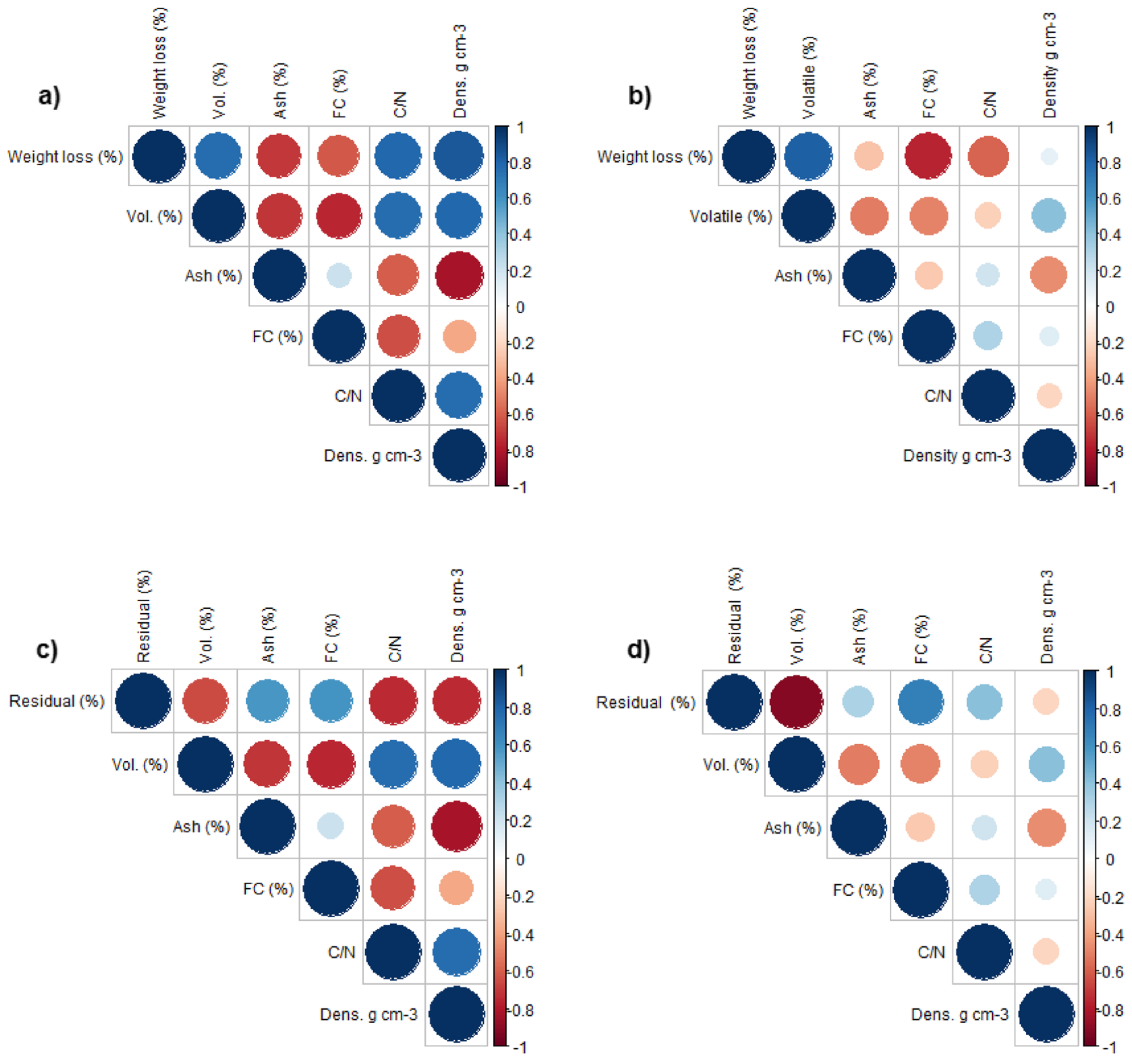
Spearman's correlation matrix between weight loss at 300–450 °C (Weight loss %), residual weight at 450 °C (Residual %), volatiles (Vol. %), ash content (Ash %), fixed carbon (FC %), C/N ratio (C/N) and density (Dens. g cm^−3^). (**a**) Weight loss of 300–450 °C from G1; (**b**) 300–450 °C weight loss of G2; (**c**) Residual weight at 450 °C of G1; (**d**) Residual weight at 450 °C of G2. Dark blue circles indicate positive correlations while dark red circles indicate negative correlations. The size of the circle indicates the strength of the correlation.

The weight loss of CWDs in the temperature range of 300–450 °C of G1 showed a positive and stronger correlation with volatile materials, C/N ratio and density. The weight loss of G2 was positively associated only with volatile materials. The residual weight at 450 °C of G1 showed a positive correlation with ash and fixed carbon content, while G2 was also positively correlated with these variables plus the C/N ratio (Fig. 4).

## Discussion

### Physical and chemical properties of CWDs

The decomposition of CWDs in forest ecosystems is a crucial pathway for nutrients return to soil^59^. During this process, CWDs on the forest floor undergo different transformations in their chemical and physical properties, such as a reduction in density, an increase in water content, accumulation of nutrients and lignin, and a reduction in pH^60^. Most of these physicochemical transformations were observed in this study.

The results indicated a reduction of the average apparent density in function of the increase of CWDs decomposition (Table 2). This reduction in density can be explained by the weight loss due to wood-decomposing microorganisms’ action^61^. Previous studies have also found this pattern of data behavior for CWDs density^37–39,50^.

The decomposition process had less impact on Carbon (C) concentrations, which did not show great variations with the decay class of CWDs increase (Table 3). The higher concentration of lignin in CWDs may be one of the factors limiting the C degradation, since it’s a large and complex structure, thus, difficult to decompose^37,62–64^. In addition, this wood constituent can also compromise the degradation of cellulose and hemicellulose when incorporated in large amounts into cell wall structures^26^.

Nitrogen (N) contents also showed low variation with increasing decay classes of CWDs (Table 3). However, this behavior was not expected since this nutrient tends to accumulate over the years due to the fixation and translocation of this chemical element from the soil to CWDs by heterotrophic microorganisms^1,65–67^. Besides, the increase in structural bonds between nitrogen and elements more resistant to degradation such as lignin, aromatic and phenolic compounds may also favor N accumulation during the wood decomposition process^68^.

The C and N contents found in our study resulted in lower C/N ratio values for the more decomposed CWDs (Table 3). This pattern of declining C/N ratio was also found in studies conducted in tropical and subtropical forests^37,38,69,70^. A low C/N ratio of CWDs indicates a greater potential for wood decomposition causing these materials to remain a shorter time in the forest ecosystem^71,72^.

### Immediate chemical analysis

The results indicated a tendency of volatile materials reduction with the CWDs decomposition (Table 4). This reduction in volatile materials may be correlated with the degradation of holocellulose and the decrease in extractive contents^73^. During the wood decomposition process, holocelluloses are readily degraded by decomposing microorganisms due to the greater ease of breaking down their structures compared to other compounds, such as lignin^74^. In the case of extractive contents, the reduction in their concentration occurs due to processes such as enzymatic deactivation, auto-oxidation, microbial degradation or by leaching^1^.

The ash contents showed a contrary trend to the volatiles, increasing their concentration with CWDs decay class increase (Table 4). These results suggest that CWDs accumulate inorganic nutrients such as potassium, calcium, magnesium and silicon in their composition as they lose weight and carbon through the decomposition process^38,66,75^. In addition, contamination by soil residues may also contribute to the higher ash content in the most decomposed CWDs. However, there is no way to measure the impacts of this contamination on our results.

Fixed carbon was not related to CWDs decay class in this study (Table 4). The fact that this component of immediate chemical analysis is obtained by difference may have influenced these results. However, fixed carbon presented values inversely proportional to volatile materials, indicating a negative correlation with holocelluloses and a positive correlation with lignin^37,68^.

### Thermogravimetric analysis

Thermogravimetric analyzes indicated that samples with a high degree of decomposition have high residual mass and low weight loss. This pattern can be explained by the greater loss of carbon in the forest ecosystem due to the respiration of microorganisms, photodegradation and leaching^76,77^. Thermogravimetric analysis also indicated that hemicellulose decomposition occurs at lower temperatures (225–275 °C). The decomposition of hemicellulose in this temperature range is related to its amorphous chemical structure^78^, composed of sugars such as pentoses and hexoses^79,80^. Thermal degradation of cellulose occurs at 275–375 °C due to its crystallinity^81^. Lignin generally begins to decompose at lower temperatures^82^, but it is the last wood compound to fully decompose due to its cross-linked structure and high molecular weight^79^.

Furthermore, TG/DTG curves indicated different behaviors for the CWDs in the different size and decay classes. The longer lengths of the DTG curve, such observed in the first decay classes (G1), indicate higher concentrations of holocelluloses in samples with larger diameters. In addition, the lower residual weight for samples with larger diameters demonstrate that the rate of decomposition in the forest ecosystem is higher for smaller CWDs^77^. The distinct weight losses, such observed in the second (G2) and third (G3) decay classes, can be explained by the wide variety of tree species present in the forest. Less weight loss and greater residual weight, as observed in the last decomposition class (G4), demonstrate that the structural components of wood have already been significantly degraded in the forest ecosystem. Comparing the four decay classes, the DTG peaks were shifted to higher temperatures with increasing decay class, which is related to the removal of extractives. In fact, as the decomposition process occurs, the extractives are released and the temperature of thermal degradation of cellulose and hemicellulose shifts to higher temperatures^83^.

Analysis of variance and Tukey's test indicated statistical difference only for the averages of weight loss in the last temperature range (300–450 °C) and for the averages of residual weight at 450 °C, differing between the decay classes 1, 2 and 3 (Group 1) of decay class 4 (Group 2) (Fig. 3). The difference between Groups 1 and 2 can be explained mainly by the apparent density and the C/N ratio, which are highly impacted variables throughout the CWD decomposition process (Fig. 4). In this way, the determination of physical and chemical properties, together with immediate and thermogravimetric analysis, can be considered an important tool to study the effects of CWDs decomposition process, defining with greater precision the classes of CWDs decomposition.

Our results narrowed the existing uncertainties in understanding the natural decomposition dynamics of CWDs. Although our study area is limited, the new guidelines used to determine the physical–chemical parameters of CWDs will serve as a basis for the sustainable management of this component and for the refinement of international reports that aim to quantify the carbon balance in forest ecosystems. Future research should focus on performing these analyzes at the species level, since this factor has a great influence on the physicochemical properties of CWDs^38,39^. Furthermore, it is recommended to carry out the microbiota characterization present in the soil and in the CWDs to distinguish the types of microorganisms and their abilities to degrade each constituent of the wood^1,84^. Finally, climatic factors such as temperature and humidity must also be considered as they influence the activity and selection of these microorganisms in the forest ecosystem^27,85^.

## Conclusions

The apparent density of CWDs is affected by the natural process of decomposition while the carbon and nitrogen contents are less impacted by this process. The C/N ratio decreased directly with the decomposition class of the CWDs. The size classes of CWDs are not relevant for determining these properties. The physical–chemical properties must be always quantified according to the decay classes of the CWDs due to the diversity of species and the climatic conditions of each forest ecosystem.

The CWDs structural chemical composition is affected by decomposition, resulting in loss of holocelluloses and extractives and an increase in lignin and ash concentration throughout this process. The weight loss is greater for the less decomposed CWDs and the residual weight is greater for the more decomposed ones. The smallest diameter classes have less weight loss and greater residual weight. The use of immediate chemical and thermogravimetric analysis removes the subjectivity to classify CWDs decomposition stages, reducing the number of tests to determine the physical and chemical properties of CWDs.

Our results contribute to a better understanding of the decomposition dynamics of CWDs and provide important information about their ecological role. The technical guidelines presented in this study should be applied and improved in other forest ecosystems around the world to increase the accuracy of scientific studies and international reports focused on the carbon cycle of these materials.

## Data Availability

The datasets generated during and/or analysed during the current study are available from the corresponding author on reasonable request.

## References

[1] Harmon, M. E. *et al.* Ecology of Coarse Woody Debris in Temperate Ecosystems. in *Advances in Ecological Research* vol. 15 133–302 (Elsevier, 1986).

[2] Christensen M (2005). Dead wood in European beech (Fagus sylvatica) forest reserves. For. Ecol. Manag..

[3] Campbell JL (2019). Estimating uncertainty in the volume and carbon storage of downed coarse woody debris. Ecol. Appl..

[4] Araujo LS, Komonen A, Lopes-Andrade C (2015). Influences of landscape structure on diversity of beetles associated with bracket fungi in Brazilian Atlantic Forest. Biol. Conserv..

[5] Seibold S (2015). Experimental studies of dead-wood biodiversity-a review identifying global gaps in knowledge. Biol. Conserv..

[6] Thibault M, Moreau G (2016). Enhancing bark- and wood-boring beetle colonization and survival in vertical deadwood during thinning entries. J. Insect Conserv..

[7] Pan Y (2011). A large and persistent carbon sink in the World’s forests. Science.

[8] Harmon ME (2021). The role of woody detritus in biogeochemical cycles: Past, present, and future. Biogeochemistry.

[9] Martin AR, Domke GM, Doraisami M, Thomas SC (2021). Carbon fractions in the world’s dead wood. Nat. Commun..

[10] Köhl M (2015). Changes in forest production, biomass and carbon: Results from the 2015 UN FAO Global Forest Resource Assessment. For. Ecol. Manag..

[11] McDowell N (2018). Drivers and mechanisms of tree mortality in moist tropical forests. New Phytol..

[12] Scaranello MAS (2019). Estimation of coarse dead wood stocks in intact and degraded forests in the Brazilian Amazon using airborne lidar. Biogeosciences.

[13] Venter O (2016). Sixteen years of change in the global terrestrial human footprint and implications for biodiversity conservation. Nat. Commun..

[14] Diniz MF, Coelho MTP, de Sousa FG, Hasui É, Loyola R (2021). The underestimated role of small fragments for carnivore dispersal in the Atlantic Forest. Perspect. Ecol. Conserv..

[15] Chambers JQ (2013). The steady-state mosaic of disturbance and succession across an old-growth Central Amazon forest landscape. Proc. Natl. Acad. Sci..

[16] da Rocha SJSS (2020). Drought effects on carbon dynamics of trees in a secondary Atlantic Forest. For. Ecol. Manag..

[17] da Rocha SJSS (2018). Artificial neural networks: Modeling tree survival and mortality in the Atlantic Forest biome in Brazil. Sci. Total Environ..

[18] Souza CR (2021). Long-term ecological trends of small secondary forests of the atlantic forest hotspot: A 30-year study case. For. Ecol. Manag..

[19] Yizhao C (2015). The role of residence time in diagnostic models of global carbon storage capacity: Model decomposition based on a traceable scheme. Sci. Rep..

[20] Barbosa RI (2017). Decomposition rates of coarse woody debris in undisturbed Amazonian seasonally flooded and unflooded forests in the Rio Negro-Rio Branco Basin in Roraima. Brazil. For. Ecol. Manag..

[21] Brienen RJW (2015). Long-term decline of the Amazon carbon sink. Nature.

[22] Bonal D, Burban B, Stahl C, Wagner F, Hérault B (2016). The response of tropical rainforests to drought—lessons from recent research and future prospects. Ann. For. Sci..

[23] Harmon ME (2020). Release of coarse woody detritus-related carbon: A synthesis across forest biomes. Carbon Balance Manag..

[24] Zhou L, Dai L, Gu H, Zhong L (2007). Review on the decomposition and influence factors of coarse woody debris in forest ecosystem. J. For. Res..

[25] Russell MB (2015). Quantifying carbon stores and decomposition in dead wood: A review. For. Ecol. Manag..

[26] Magnússon RÍ, Tietema A, Cornelissen JHC, Hefting MM, Kalbitz K (2016). Tamm review: Sequestration of carbon from coarse woody debris in forest soils. For. Ecol. Manag..

[27] Bradford MA, Berg B, Maynard DS, Wieder WR, Wood SA (2016). Understanding the dominant controls on litter decomposition. J. Ecol..

[28] Fioretto A, Di Nardo C, Papa S, Fuggi A (2005). Lignin and cellulose degradation and nitrogen dynamics during decomposition of three leaf litter species in a Mediterranean ecosystem. Soil Biol. Biochem..

[29] Colodette, J. L. & Gomes, F. J. B. *Branqueamento de polpa celulósica: Da produção da polpa marrom ao produto acabado*. (Editora UFV, 2015).

[30] Martínez AT (2005). Biodegradation of lignocellulosics: Microbial, chemical, and enzymatic aspects of the fungal attack of lignin. Int. Microbiol. Off. J. Span. Soc. Microbiol..

[31] Fukasawa Y, Osono T, Takeda H (2009). Dynamics of physicochemical properties and occurrence of fungal fruit bodies during decomposition of coarse woody debris of *Fagus crenata*. J. For. Res..

[32] Klotzbücher T, Kaiser K, Guggenberger G, Gatzek C, Kalbitz K (2011). A new conceptual model for the fate of lignin in decomposing plant litter. Ecology.

[33] Strukelj M (2013). Chemical transformations in downed logs and snags of mixed boreal species during decomposition. Can. J. For. Res..

[34] Mori S (2014). Effect of wood density and water permeability on wood decomposition rates of 32 Bornean rainforest trees. J. Plant Ecol..

[35] Pietsch KA (2014). Global relationship of wood and leaf litter decomposability: The role of functional traits within and across plant organs: Global relationship of wood and leaf litter decomposability. Glob. Ecol. Biogeogr..

[36] Chambers JQ, Higuchi N, Schimel JP, Ferreira LV, Melack JM (2000). Decomposition and carbon cycling of dead trees in tropical forests of the central Amazon. Oecologia.

[37] Meriem S, Tjitrosoedirjo S, Kotowska MM, Hertel D, Triadiati T (2016). Carbon and nitrogen stocks in dead wood of tropical lowland forests as dependent on wood decay stages and land-use intensity. Ann. For. Res..

[38] Chao K-J (2017). Carbon concentration declines with decay class in tropical forest woody debris. For. Ecol. Manag..

[39] Moreira AB, Gregoire TG, do Couto HTZ (2019). Wood density and carbon concentration of coarse woody debris in native forests, Brazil. For. Ecosyst..

[40] Bani A (2018). The role of microbial community in the decomposition of leaf litter and deadwood. Appl. Soil Ecol..

[41] Food and Agriculture Organization-FAO. Global Forest Resources Assessment 2020: Main Report. (2020).

[42] Rondeux J (2012). Assessing deadwood using harmonized national forest inventory data. For. Sci..

[43] Universidade Federal de Viçosa-UFV. *Departamento de Engenharia Agrícola. Estação Climatológica Principal de Viçosa. Boletim Meteorológico*. (2021).

[44] Ferreira Junior, W. G., Schaefer, C. E. G. R. & Silva, A. F. Uma visão pedogeomorfológica sobre as formações florestais da Mata Atlântica. in *Ecologia de Florestas Tropicais do Brasil* 141–174 (Editora UFV, 2012).

[45] QGIS.org. QGIS Geographic Information System. QGIS Association. (2020).

[46] Instituto Brasileiro de Geografia e Estatística-IBGE. *Manual técnico da vegetação brasileira*. (2012).

[47] Brasil. *Resolução CONAMA N*^*o*^* 392, de 25 de junho de 2007: Definição de vegetação primária e secundária de regeneração de Mata Atlântica no Estado de Minas Gerais.* (2007).

[48] Harmon ME, Whigham DF, Sexton J, Olmsted I (1995). Decomposition and mass of woody detritus in the dry tropical forests of the Northeastern Yucatan Peninsula, Mexico. Biotropica.

[49] Keller M, Palace M, Asner GP, Pereira R, Silva JNM (2004). Coarse woody debris in undisturbed and logged forests in the eastern Brazilian Amazon: Coarse Woody Debris in the Eastern Amazon. Glob. Change Biol..

[50] Villanova PH (2019). Necromass carbon stock in a secondary atlantic forest fragment in Brazil. Forests.

[51] Vital, B. R. *Boletim Técnico: Métodos de Determinação de Densidade da Madeira*. (Sociedade de Investigações Florestais, 1984).

[52] Associação Brasileira de Normas Técnicas-ABNT. *Normas Técnicas NBR 11941: Madeira-determinação da densidade básica.* (2003).

[53] Standard ASTM. *Standard Test Method for Chemical Analysis of Wood Charcoal*. (ASTM International, 2009).

[54] Lana AQ, Salles TT, de Carneiro ACO, Cardoso MT, Teixeira RU (2016). Comparison of procedures for immediate chemical analysis of charcoal. Rev. Árvore.

[55] Choi Y-K, Kan E (2019). Effects of pyrolysis temperature on the physicochemical properties of alfalfa-derived biochar for the adsorption of bisphenol A and sulfamethoxazole in water. Chemosphere.

[56] Hauke J, Kossowski T (2011). Comparison of values of Pearson’s and Spearman’s correlation coefficients on the same sets of data. QUAGEO.

[57] Puth M-T, Neuhäuser M, Ruxton GD (2015). Effective use of Spearman’s and Kendall’s correlation coefficients for association between two measured traits. Anim. Behav..

[58] R Core Team. R: A language and environment for statistical computing. (2020).

[59] Krishna MP, Mohan M (2017). Litter decomposition in forest ecosystems: A review. Energy Ecol. Environ..

[60] Fukasawa Y, Matsuoka S (2015). Communities of wood-inhabiting fungi in dead pine logs along a geographical gradient in Japan. Fungal Ecol..

[61] Schilling JS, Kaffenberger JT, Liew FJ, Song Z (2015). Signature wood modifications reveal decomposer community history. PLoS ONE.

[62] Freschet GT, Weedon JT, Aerts R, van Hal JR, Cornelissen JHC (2012). Interspecific differences in wood decay rates: Insights from a new short-term method to study long-term wood decomposition: New method to assess wood decay dynamics and rates. J. Ecol..

[63] Harmon ME, Fasth B, Woodall CW, Sexton J (2013). Carbon concentration of standing and downed woody detritus: Effects of tree taxa, decay class, position, and tissue type. For. Ecol. Manag..

[64] Demuner IF, Colodette JL, Demuner AJ, Jardim CM (2019). Biorefinery review: Wide-reaching products through kraft lignin. BioResources.

[65] Philpott TJ, Prescott CE, Chapman WK, Grayston SJ (2014). Nitrogen translocation and accumulation by a cord-forming fungus (Hypholoma fasciculare) into simulated woody debris. For. Ecol. Manag..

[66] Foudyl-Bey S, Brais S, Drouin P (2016). Litter heterogeneity modulates fungal activity, C mineralization and N retention in the boreal forest floor. Soil Biol. Biochem..

[67] Rinne-Garmston KT (2019). Carbon flux from decomposing wood and its dependency on temperature, wood N _2_ fixation rate, moisture and fungal composition in a Norway spruce forest. Glob. Change Biol..

[68] Hishinuma T, Osono T, Fukasawa Y, Azuma J, Takeda H (2015). Application of 13C NMR spectroscopy to characterize organic chemical components of decomposing coarse woody debris from different climatic regions. Ann. For. Res..

[69] Yang F-F (2010). Dynamics of coarse woody debris and decomposition rates in an old-growth forest in lower tropical China. For. Ecol. Manag..

[70] Fujisaki K (2015). Decomposition kinetics and organic geochemistry of woody debris in a ferralsol in a humid tropical climate: Decomposition of woody debris. Eur. J. Soil Sci..

[71] Jacob M, Viedenz K, Polle A, Thomas FM (2010). Leaf litter decomposition in temperate deciduous forest stands with a decreasing fraction of beech (Fagus sylvatica). Oecologia.

[72] Purahong W (2015). Effects of forest management practices in temperate beech forests on bacterial and fungal communities involved in leaf litter degradation. Microb. Ecol..

[73] Stefanidis SD (2014). A study of lignocellulosic biomass pyrolysis via the pyrolysis of cellulose, hemicellulose and lignin. J. Anal. Appl. Pyrolysis.

[74] Bonanomi G (2013). Litter quality assessed by solid state 13C NMR spectroscopy predicts decay rate better than C/N and Lignin/N ratios. Soil Biol. Biochem..

[75] Morris D, Wiebe S, Luckai N, Reid D (2015). Nutrient retention and leaching potential of coarse wood bolts collected from logged and burned upland boreal sites: A greenhouse misting experiment. Boreal Environ. Res..

[76] Kahl T, Mund M, Bauhus J, Schulze E-D (2012). Dissolved organic carbon from European beech logs: Patterns of input to and retention by surface soil. Écoscience.

[77] Herrmann S, Kahl T, Bauhus J (2015). Decomposition dynamics of coarse woody debris of three important central European tree species. For. Ecosyst..

[78] Hill CAS (2006). Wood Modification: Chemical, Thermal and Other Processes.

[79] Yang H (2006). In-depth investigation of biomass pyrolysis based on three major components: Hemicellulose, cellulose and lignin. Energy Fuels.

[80] Stelte W (2011). A study of bonding and failure mechanisms in fuel pellets from different biomass resources. Biomass Bioenergy.

[81] Garcia-Maraver A, Salvachúa D, Martínez MJ, Diaz LF, Zamorano M (2013). Analysis of the relation between the cellulose, hemicellulose and lignin content and the thermal behavior of residual biomass from olive trees. Waste Manag..

[82] Nassar MM, MacKay GDM (1984). Mechanism of thermal decomposition of lignin. Wood Fiber Sci..

[83] Poletto M (2016). Effect of extractive content on the thermal stability of two wood species from Brazil. Maderas Cienc. Tecnol..

[84] Cornwell WK (2009). Plant traits and wood fates across the globe: Rotted, burned, or consumed?. Glob. Change Biol..

[85] De la Cruz FB, Yelle DJ, Gracz HS, Barlaz MA (2014). Chemical changes during anaerobic decomposition of hardwood, softwood, and old newsprint under mesophilic and thermophilic conditions. J. Agric. Food Chem..

